# Protein–nucleic acid binding site prediction using interpretable Kolmogorov–Arnold networks with hypergraph representation learning

**DOI:** 10.1093/bioinformatics/btag433

**Published:** 2026-06-20

**Authors:** Yangfeng Zhu, Guicong Sun, Weimin Zhu, Yongxian Fan, Zeheng Wu, Xianchen Zheng, Xiaoyong Pan

**Affiliations:** School of Computer Science and Information Security, Guilin University of Electronic Technology, Guilin 541004, China; School of Computer Science and Information Security, Guilin University of Electronic Technology, Guilin 541004, China; Institute of Image Processing and Pattern Recognition, Shanghai Jiao Tong University, and Key Laboratory of System Control and Information Processing, Ministry of Education of China, Shanghai 200240, China; School of Computer Science and Information Security, Guilin University of Electronic Technology, Guilin 541004, China; School of Computer Science and Information Security, Guilin University of Electronic Technology, Guilin 541004, China; School of Computer Science and Information Security, Guilin University of Electronic Technology, Guilin 541004, China; Institute of Image Processing and Pattern Recognition, Shanghai Jiao Tong University, and Key Laboratory of System Control and Information Processing, Ministry of Education of China, Shanghai 200240, China

## Abstract

**Motivation:**

In recent years, protein language models (pLMs) and graph neural networks (GNNs) have demonstrated powerful expressive and reasoning capabilities in modeling protein-RNA/DNA interactions. However, existing methods, which use simple graphs to describe the relationships between residues, struggle to effectively capture the high-order, multi-body residue interactions present in protein–nucleic acid complex structures. In fact, spatially continuous but sequence-wise discontinuous residues often cooperatively determine nucleic acid binding capacity.

**Results:**

In this study, we present IKANbind, a computational approach that combines hypergraph representation learning and interpretable Kolmogorov–Arnold Networks (KANs), for identifying nucleic acid binding residues (NBRs) in proteins. By combining the advantages of pLM, hypergraph neural networks and symbolic KAN, IKANbind outperforms existing methods on multiple NBR benchmark datasets. We also demonstrated that the pLM used in IKANbind can implicitly learn the physicochemical properties of binding residues, such as charge and hydrophobicity. In addition, the symbolic KAN, which uses a unique weighted mechanism of decomposable basis functions, can accurately identify the features with the greatest contribution to NBR recognition. We found that polarity and charge make greater contributions to NBR prediction than other physicochemical properties or evolutionary information. Finally, IKANbind achieves promising performance when extended to other ligand-binding residue prediction tasks.

**Availability and implementation:**

IKANbind is freely available at https://github.com/yangfengzhuguet/IKANBind.

## 1 Introduction

Protein–nucleic acid interactions are crucial for gene expression, genetic information transfer, and the maintenance of cellular homeostasis (He *et al.* 2023, Basu *et al.* 2024). Identifying the interaction patterns of nucleic acid binding residues (NBRs) in proteins is significant for understanding disease mechanisms (Tao *et al.* 2024) and promoting drug discovery (Schmidtke and Barril 2010). Although early experiment-based methods, such as X-ray crystallography (Kim *et al.* 2022) and cryo-electron microscopy (Yu *et al.* 2022), can resolve protein–nucleic acid complex structures with a high resolution, their high cost and low throughput limit their application in large-scale studies. To overcome these limitations, many computational methods have been proposed to predict NBRs in proteins. Such computational methods can be categorized into sequence-based and structure-based approaches. Sequence-based methods, such as CLAPE (Liu and Tian 2023), ULDNA (Zhu *et al.* 2024), DNAPred (Yan and Kurgan 2017), TargetS (Yu *et al.* 2013), SVMnuc (Su *et al.* 2019), SCRIBER (Zhang and Kurgan 2019), NCBRPred (Zhang *et al.* 2021), and TargetDNA (Hu *et al.* 2017), typically rely on protein sequences to extract diverse features (e.g. evolutionary information, physicochemical properties, and sequence alignment information) to learn local patterns associated with NBRs. For instance, NCBRPred uses bidirectional gated recurrent units to capture global dependencies among residues for predicting NBRs. In contrast, structure-driven methods, such as GraphBind (Xia *et al.* 2021), aaRNA (Li *et al.* 2014), GraphSite (Yuan *et al.* 2022), NucBind (Su *et al.* 2019), COACH-D (Wu *et al.* 2018), DNABind (Liu and Hu 2013), and NABind (Jiang *et al.* 2023), integrate available structural information to model spatial dependencies among residues. For example, GraphBind encodes protein structures as graphs by graph neural networks (GNNs) to predict NBRs. Remarkably, GraphBind has provided significant inspiration for subsequent approaches combining protein language models (pLMs) with GNNs. Although structure-driven methods achieve a higher accuracy, they highly rely on high-quality structures.

The recent breakthrough of AlphaFold2 (Jumper *et al.* 2021, Varadi *et al.* 2022) demonstrates that protein 3D structures can be predicted with high accuracy solely from sequence information, which provides a viable alternative for replacing experimental structures with predicted ones in protein-related tasks. GraphSite has successfully applied AlphaFold2-predicted structures to the protein–DNA binding site prediction task. However, AlphaFold2, which relies on multiple sequence alignment and template retrieval, requires high computational and storage costs. The success of AlphaFold2 not only confirms the potential of deep neural networks in structural modeling but also promotes the rapid development of Transformer-based pLMs (Rives *et al.* 2021, Elnaggar *et al.* 2022, Lin *et al.* 2023, Hayes *et al.* 2025). Such pLMs, through unsupervised pre-training on large-scale protein sequences, can capture rich semantic and structural information, and have been widely used in predicting protein–protein interactions (PPI; Shen *et al.* 2025) and protein-ligand binding sites (Guan *et al.* 2025). In recent years, combining pLMs with GNNs has gained widespread attention, such as ATMGBs (Li *et al.* 2025), EquiPNAS (Roche *et al.* 2024), EGPDI (Zheng *et al.* 2024), GLMSite (Song *et al.* 2023), and GeSite (Zeng *et al.* 2025). Among these, GNNs with E(3)-equivariant graph neural networks [EGNNs (Satorras *et al.* 2021)] exhibit superior performance in structural modeling. For example, EquiPNAS encodes proteins by combining pLM embeddings with EGNNs to capture complex geometric relationships between residues. However, these methods are generally based on the simple graph structures, making it difficult to effectively model higher-order cooperative interactions among multiple spatially adjacent residues, which cooperatively determine nucleic acid binding capacity in proteins.

Although pLM-based methods have made significant progress in protein-related tasks, they typically utilize multilayer perceptrons (MLPs) for making final predictions, where the relationship between input features and outputs is often concealed within complex weight matrices and nonlinear activation functions, making them difficult to interpret directly. The recently proposed Kolmogorov–Arnold Network (KAN; Liu *et al.* 2024), as a potential alternative to MLPs, shows promising prospects for enhancing model expressive power and mathematical interpretability. KAN, which replaces traditional activation functions with composable B-spline basis functions, can explicitly map the relationship between inputs and outputs. KAN has achieved promising performance across multiple prediction tasks, including drug–target interaction (Fu *et al.* 2026), enhancer–promoter interaction (Zhang *et al.* 2025), and miRNA–disease association prediction (Zhu *et al.* 2025). Although KAN provides mathematical interpretability, B-spline functions lack symbolic readability and are difficult to fit the distribution of different features.

Here, we present a deep hypergraph neural network framework called IKANbind to improve protein–nucleic acid binding site identification. To represent the interactions among multiple residues, we first construct a hypergraph based on the 3D structure of the protein. The node feature space comprises pLM embeddings and traditional hand-crafted features. We then use hypergraph convolutional networks (HGCNs) and hypergraph attention networks (HGATs) to learn the latent binding patterns of NBRs. Our ablation experiments demonstrate that the pLM in IKANbind not only reduces reliance on evolutionary information but also implicitly encodes the physicochemical properties of amino acids. In addition, symbolic KAN, which apply unique basis function weight assignment mechanism, can accurately identify the latent features that contribute the most to the NBR task. We found that the polarity and charge properties of amino acids are more important for NBR prediction than other physicochemical properties and evolutionary information. Finally, IKANbind exhibits excellent performance in other ligand-binding residue prediction tasks.

## 2 Materials and methods

### 2.1 The overview of IKANbind

**Figure 1:**
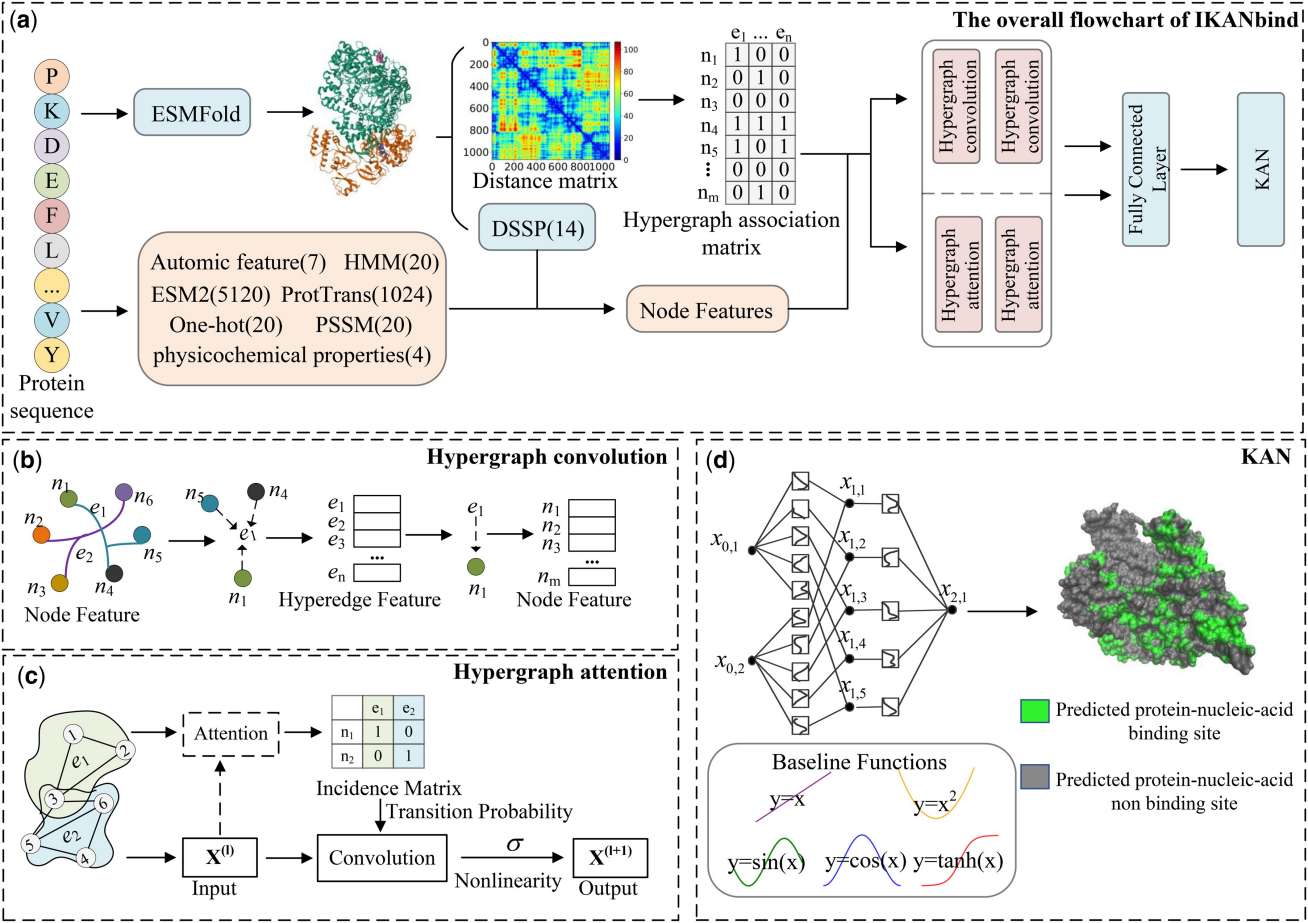
The overall architecture of IKANbind. (a) Workflow of IKANbind. The protein sequence is input into ESMFold and pLMs (ESM2, protTrans), respectively, to obtain the protein 3D structure and pLM embedding. The 3D structure is used to construct the hypergraph, where the node feature space includes pLM embeddings and handcrafted features. Then, high-order protein representation is extracted through hypergraph convolution and hypergraph attention. Finally, the binding sites are predicted by the KAN classifier. (b) The architecture of hypergraph convolution. Node features are propagated in a node-hyperedge-node manner to learn global contextual information. The hyperedge weight matrix is computed based on the Jaccard similarity and the structural similarity of shared nodes. (c) The architecture of hypergraph attention. Hypergraph attention is computed based on hypergraph convolution. It computes the dynamic transition matrix by learning a dynamic correlation matrix, which transforms the original binary associations into continuous attention weights. (d) The architecture of KAN. KAN, which employs basis functions with explicit mathematical meanings (e.g. tanh(x), sin(x)), can capture different types of features, such as saturation and periodicity.

The overall workflow of IKANbind is illustrated in Fig. 1a. First, the protein sequence is input into ESMFold and pLMs (ESM2 and protTrans) to obtain the 3D structure and pLM embeddings of the protein. Then, a hypergraph is constructed based on the centroid distances between residues in the 3D structure to describe the interactions among multiple residues. Subsequently, hypergraph convolution (Fig. 1b) and hypergraph attention (Fig. 1c) are used to learn the higher-order representation of the protein. Hypergraph convolution aggregates and propagates features in a node-hyperedge-node fashion and hypergraph attention learns the associated dynamic transition matrix to better capture the intrinsic relationships between nodes. Finally, the KAN classifier (Fig. 1d) is used to predict the binding sites of the nucleic acids. The symbolic KAN, which uses a set of functions with explicit mathematical meaning, can simultaneously capture diverse features such as saturation, periodicity, linearity, and nonlinearity associated with NBRs. To evaluate the performance of IKANbind, we evaluate it on published benchmark test sets from GLMSite (Song *et al.* 2023) and a strictly non-redundant independent test set based on time split against baseline methods. Moreover, we conducted a performance comparison between IKANbind and AlphaFold3, which enables protein-RNA/DNA complex prediction. We calculate the accuracy (ACC), precision (PRE), recall (REC), F1-score (F1), Matthews correlation coefficient (MCC), area under the receiver operating characteristic curve (AUC), and area under the precision–recall curve (AUPR), as given in Section 1, available as supplementary data at *Bioinformatics* online. In addition to AUC and AUPR, the other metrics were calculated based on a threshold determined by maximizing the MCC, which converts the predicted NBR probabilities into binary labels.

### 2.2 Benchmark datasets

We use the training sets (DNA-735-Train and RNA-577-Train) and test sets (DNA-180-Test and RNA-93-Test) from GLMSite (Song *et al.* 2023) to evaluate the performance of protein–nucleic acid binding site prediction methods. These datasets are originally downloaded from the public BioLip database (released on 20 March 2022) and pre-processed using MMseqs2 (Steinegger and Söding 2017) to remove chains with less than 30% sequence identity. The DNA-binding residue training set (DNA-735-Train) was released before 18 December 2019, whereas the test set (DNA-180-Test) was released after that date. The RNA-binding residue training set (RNA-577-Train) was released before 19 June 2019, whereas the test set (RNA-142-Test) was released after that date. The binding residues are defined based on the minimum atomic distance to nucleic acids: if this distance minus 0.5 Å is less than the sum of the van der Waals’ radius of the two nearest atoms, the residue is considered a NBR.

In addition, we construct a strictly non-redundant independent test set to further evaluate the generalization of IKANbind. Specifically, we downloaded 83 044 non-redundant protein-ligand interaction entries from the BioLiP database (released on 30 July 2025), which includes 4042.

DNA-binding protein entries and 9107 RNA-binding protein entries. We set the deposition dates for the DNA- and RNA-binding test sets to after 18 December 2019 and 19 June 2019. We then used MMseqs to remove protein chains with sequence similarity over 30% to any chain in the benchmark training set, and further de-redunded the DNA/RNA binding test set internally using MMseqs with 100% sequence identity and 80% coverage thresholds. Additionally, we further filtered protein entries with multi-chain identifiers (such as 9G6K_LY) using the Q-BioLip (Wei *et al.* 2024) database. Finally, we obtained 112 DNA-binding proteins and 93 RNA-binding proteins as the DNA- and RNA-binding independent test set. The details of the datasets can be seen in Table 1.

**Table 1:** Summary of benchmark datasets.

Type	Dataset	Proteins	Binding residues	Nonbinding residues	Ratio
DNA	DNA-735-Train	735	18 611	178 125	0.104
	DNA-180-Test	180	4255	60 964	0.070
	DNA-112-Test	112	1986	44 924	0.044
RNA	RNA-577-Train	577	18 564	143 019	0.130
	RNA-142-Test	142	4481	53 233	0.084
	RNA-93-Test	93	3239	44 288	0.073

Ratio = binding residues/non-binding residues.

### 2.3 Hypergraph representation learning

#### 2.3.1 Hypergraph construction

In this study, the nucleic acid-binding residue prediction task is treated as a node classification problem. The protein structure is represented as a hypergraph, which is defined as G=(X,E,H). $X={\left\{x_{i}\right\}}_{i=1}^{N}$ and $x_{i}$ denote the node (residue) feature matrix and the feature vector of node i, respectively, where N is the length of the protein sequence. The detailed information for node features can be seen in Table 1, available as supplementary data at *Bioinformatics* online. $E={\left\{e_{j}\right\}}_{j=1}^{M}$ represents the set of hyperedges, where each hyperedge comprises multiple spatially adjacent nodes. Specifically, for each node i, a hyperedge $e_{j}$ is constructed centered on it. If the centroid distance between the node j and i is less than 8 Å, the node j is added to the hyperedge. The relationship between nodes and hyperedges is represented by the incidence matrix H, which is defined below:


(1) $$H_{ij}=\{\begin{array}{c} 1,\;\;\mathrm{if}\;x_{i}\in e_{j}\\ 0,\;\;\mathrm{if}\;x_{i}\notin e_{j} \end{array}$$

#### 2.3.2 Hypergraph convolutional network

Traditional simple graphs are limited to pairwise relationships between nodes, which makes it difficult to handle interactions among multiple entities in complex biological systems. As a generalization of simple graphs, hypergraphs allow an edge to connect more than two nodes, which provides a more flexible structure. Based on the advantages of hypergraphs, the hypergraph convolutional network (Bai *et al.* 2021) can achieve high-order feature interactions between multiple nodes in a convolution operation through a bidirectional information propagation mechanism between nodes and hyperedges. The propagation mechanism of the HGCN layer is defined below:


(2) $$X^{\left(l+1\right)}=\text{ReLU}(D_{v}^{-1/2}HWD_{e}^{-1}H^{T}D_{v}^{-1/2}X^{\left(l\right)}\Theta^{\left(l\right)})$$

where $D_{v}$ and $D_{e}$ are the degree matrices of nodes and hyperedges, respectively. $\Theta^{\left(l\right)}$ is a learnable parameter matrix. Furthermore, we consider both the Jaccard similarity of their node sets and the structural similarity between shared nodes for hyperedges $e_{i}$ and $e_{j}$ in the hyperedge weight matrix W. Specifically, let $s_{i}$ and $s_{j}$ represent the node sets contained in hyperedges $e_{i}$ and $e_{j}$. The Jaccard similarity is defined as:


(3) $$\text{Jaccard}(e_{i},e_{j})=\frac{\left|s_{i}\cap s_{j}\right|}{\left|s_{i}\cup s_{j}\right|}$$

To measure the spatial proximity of shared nodes, we define the structural similarity (StructureSim) as the Gaussian kernel mean of the side-chain centroid distances for all shared node pairs:


(4) $$\text{StructureSim}(e_{i},e_{j})=\frac{1}{\left|N\right|}\sum_{(u,v)\in N}\;\text{exp}(-\frac{d_{uv}^{2}}{2\gamma^{2}})$$

where N denotes the node intersection of hyperedges $e_{i}$ and $e_{j}$, $d_{uv}$ denotes the side chain centroid distance between nodes u and v, and γ is the Gaussian kernel bandwidth. Finally, the weight between hyperedges is defined as a weighted combination of the two:


(5) $$W_{ij}=\alpha \cdot \text{Jaccard}(e_{i},e_{j})+(1-\alpha )\cdot \text{StructureSim}(e_{i},e_{j})$$

where α∈[0,1] is the parameter that controls the balance between the two types of similarity. To prevent the loss of original information during propagation, the self-connection weight of each hyperedge is set to one.

#### 2.3.3 Hypergraph attention networks

The HGAT (Bai *et al.* 2021) introduces an attention mechanism on top of the HGCN to better reveal the intrinsic relationships between vertices. Hypergraph convolution uses the incidence matrix H to describe the relationships between nodes and hyperedges, which struggles to reflect the importance of different connections. Therefore, we add an attention learning module into the incidence matrix H, transforming the binary incidence matrix into a continuous attention weight matrix (dynamic transition matrix), which is computed as follows:


(6) $${\tilde{H}}_{ij}=\frac{\;\text{exp}(\text{ReLU}(a^{T}\left[x_{i}\|x_{j}\right]))}{\sum_{k\in \varepsilon (i)}\;\text{exp}(\text{ReLU}(a^{T}\left[x_{i}\|x_{k}\right]))}$$

where ε(i) represents the neighborhood set of $x_{i}$ and a is a weight vector used to output a scalar similarity value. The propagation process of HGAT is as follows:


(7) $$X^{\left(l+1\right)}=\text{eLU}(D^{-1/2}\tilde{H}W_{e}B^{-1}{\tilde{H}}^{T}D^{-1/2}X^{\left(l\right)}P)$$

where D and B are the degree matrices of nodes and hyperedges, respectively, and P is the weight matrix.

### 2.4 Kolmogorov–Arnold network

KAN originates from the Kolmogorov–Arnold representation theorem (Tikhomirov 1991), which states that any multivariate continuous function $f:{\left[0,1\right]}^{n}\to R$ can be expressed as a finite sum of univariate continuous functions:


(8) $$f(x)=f(x_{1},x_{2},\ldots ,x_{n})=\sum_{q=1}^{2n+1}\Phi_{q}\left(\sum_{p=1}^{n}\psi_{q,p}(x_{p})\right)$$

where $\psi_{q,p}:[0,1]\to R$ and $\Phi_{q}:R\to R$ are continuous univariate functions. Unlike the complex weights in MLPs, KAN employs parameterizable univariate basis functions to achieve nonlinear mapping between inputs and outputs, thereby enhancing the representational capacity of the model. Although KAN has a clear mathematical expression structure, the B-spline basis functions remain abstract in terms of readability. In this study, we define a set of functions, denoted as $B=\{\;\text{sin}\;\pi *x,\;\text{cos}\;\pi *x,\tanh\;\pi *x,x,x^{2}\}$, which have explicit mathematical meaning. In addition, the π-scaling in the basis functions is used to adjust the variation density of the nonlinear function within a finite interval. Each $\psi_{q,p}(\cdot )$ is defined as a linear combination of the above basis functions:


(9) $$\psi_{q,p}(x)=\theta_{q,p,1}\;\text{sin}(\pi *x)+\theta_{q,p,2}\;\text{cos}(\pi *x)+\theta_{q,p,3}\tanh(\pi *x)+\theta_{q,p,4}x+\theta_{q,p,5}x^{2}$$

where $\theta_{q,p,m}$ is a learnable parameter. During the forward propagation of the network, the input vector $X={\left[x_{1},x_{2},\ldots ,x_{n}\right]}^{T}$ is first transformed by the univariate basis function $\psi_{q,p}(\cdot )$, then aggregated through summation and the combination function $\Phi_{q}(\cdot )$ within the hidden layer, and the final output is defined as follows:


(10) $$y=\sum_{q=1}^{2n+1}\Phi_{q}(\sum_{p=1}^{n}\sum_{m=1}^{5}\theta_{q,p,m}b_{m}(x_{p}))$$

where $b_{m}(\cdot )\in B,\;m=1,\ldots ,5$. By introducing the above mathematical functions as basis functions, the IKANbind can simultaneously capture periodic, saturated nonlinear, and linear features, thereby enhancing its nonlinear approximation capability. Furthermore, compared with B-spline basis functions, symbolic functions possess explicit mathematical expressions, which provide a clearer mathematical structure between inputs and outputs.

### 2.5 Implementation details

#### 2.5.1 Parameter settings

In the architecture of IKANbind, the HGCN module consists of two layers with a hidden dimension of 512 (with parameters α=0.5 and γ=1.0). The HGAN module consists of two layers with the same hidden dimension of 512. During training, we use the binary cross-entropy loss function. We also use the Adam optimizer with a learning rate of 1e-4 and the weight decay of 1e-5. To reduce the risk of overfitting, we apply dropout regularization to the node embeddings of the HGCN and HGAN layers, with dropout rates of 0.3 and 0.2, respectively.

#### 2.5.2 Experimental setting

We implemented IKANbind based on Python v3.8.20 and PyTorch v2.1.2. The versions of other software dependencies are provided in the environment configuration file available at the project repository (https://github.com/yangfengzhuguet/IKANBind/blob/main/environment.yml). We trained IKANbind for the DNA-735-Train dataset on an NVIDIA GeForce RTX 3090 GPU. For the RNA-577-Train dataset, training was conducted in a virtual GPU environment equipped with an AMD EPYC 9754 processor (24 cores) and a 32 GB GPU.

## 3 Results

### 3.1 IKANbind outperforms baseline methods on benchmark datasets

Table 2 presents the performance comparison of IKANbind with other baseline methods on the independent test sets. The detailed information for baseline methods can be seen in Table 2, available as supplementary data at *Bioinformatics* online. IKANbind respectively achieved improvements of 1.1% and 7.1% in AUC and AUPR on the DNA-180-Test over the second-best method (ATMGBs), and was comparable to ATMGBs on the RNA-142-Test. We observed a larger improvement in AUPR, objective metrics for imbalanced classification, suggesting that IKANbind is better at identifying positive samples in imbalanced datasets. It is noteworthy that template-based COACH-D and machine learning-based SVMnuc rely on known structures or handcrafted features, which struggle to capture higher-order relationships in protein–nucleic acid interactions effectively. In contrast, GLMSite and ATMGBs that combine pLMs with GNNs can automatically learn deep representations of sequence and structure, but are limited to pairwise residue relationships, failing to adequately capture multi-residue spatial interactions. IKANbind, which combines the strength of the pLM and hypergraph, successfully overcomes these limitations.

**Table 2:** Performance comparison of IKANbind and baseline methods on four test sets of nucleic acid binding sites.

Dataset	Method	ACC	PRE	REC	F1	MCC	AUC	AUPR
DNA-180-Test	COACH-D(a)	0.924	0.403	0.307	0.349	0.312	0.691	0.263
	NucBind(a)	0.924	0.411	0.337	0.370	0.332	0.813	0.339
	SVMnuc(a)	0.923	0.403	0.324	0.359	0.321	0.820	0.333
	DNAPred	0.909	0.411	0.357	0.382	0.334	0.824	0.399
	GraphBind(a)	0.923	0.425	0.522	0.468	0.430	0.886	0.423
	GLMSite(a)	**0.931**	**0.475**	0.539	0.505	0.469	0.905	0.503
	CLAPE(b)	0.911	0.347	0.419	0.380	0.334	0.826	0.354
	ATMGBs(b)	**0.931**	0.470	0.512	0.489	0.453	0.906	0.491
	**IKANbind**	0.923	0.434	**0.611**	**0.508**	**0.475**	**0.916**	**0.526**
DNA-112-Test	GLMSite(a)	0.948	0.415	0.547	0.472	0.450	0.913	0.446
	CLAPE(b)	0.951	0.406	0.321	0.358	0.336	0.842	0.304
	ATMGBs(b)	0.947	0.412	0.584	0.483	0.464	0.924	0.454
	EquiPNAs(a)	0.945	0.407	**0.557**	0.487	0.466	0.932	0.461
	**IKANbind**	**0.953**	**0.457**	0.535	**0.493**	**0.470**	**0.933**	**0.472**
RNA-142-Test	COACH-D(a)	**0.909**	0.273	0.106	0.152	0.128	0.542	0.153
	NucBind(a)	0.903	0.284	0.166	0.210	0.168	0.714	0.201
	SVMnuc(a)	0.901	0.274	0.162	0.204	0.161	0.719	0.193
	GraphBind(a)	0.867	0.279	0.453	0.345	0.285	0.789	0.275
	GLMSite(a)	0.895	**0.362**	0.462	**0.406**	**0.352**	0.837	0.331
	CLAPE(b)	0.864	0.253	0.383	0.305	0.239	0.751	0.221
	ATMGBs(b)	0.883	0.329	**0.483**	0.391	0.337	**0.839**	0.334
	**IKANbind**	0.893	0.350	0.445	0.392	0.337	0.837	**0.340**
RNA-93-Test	GLMSite(a)	**0.899**	0.318	0.390	0.350	0.298	0.814	0.288
	CLAPE(b)	0.878	0.235	0.329	0.274	0.213	0.753	0.215
	ATMGBs(b)	0.887	0.314	0.514	0.389	0.344	0.849	0.337
	EquiPNAs(a)	0.867	0.285	**0.595**	0.385	0.349	0.848	0.349
	IKANbind	0.889	**0.323**	0.534	**0.403**	**0.359**	**0.861**	**0.360**

(a) denotes methods based on predictive structures, (b) denotes methods based on sequences. Bold text indicates the best result among the compared methods.

To demonstrate the generalizability of IKANbind, we evaluate it on strictly independent test sets based on time split and sequence similarity. Moreover, The EGNNs introduce geometric positional information on traditional GNNs, demonstrating strong modeling capabilities in protein structure-related tasks. Therefore, we selected EquiPNAs, an EGNN-based approach, as one of the baseline methods for comparison on the strictly independent DNA-112-Test and RNA-93-Test datasets. The results show that the overall performance of EquiPNAs is comparable to IKANbind in DNA/RNA binding residue prediction tasks. Specifically, IKANbind achieved AUCs of 0.933 and 0.861, respectively, while EquiPNAs achieved 0.932 and 0.848, on DNA-112-Test and RNA-93-Test. Furthermore, we compared the per-protein GPU memory consumption during an epoch for both methods on the RNA-577-Train dataset. Figure 1, available as supplementary data at *Bioinformatics* online illustrates that EquiPNAs demands significantly more GPU memory than IKANbind during training. In contrast, IKANbind markedly reduced computational resource consumption at the single-protein level, indicating that IKANbind achieves a better balance between the predictive performance and computational efficiency. Table 3, available as supplementary data at *Bioinformatics* online reports the comparison of IKANbind with structure-based methods using experimentally determined protein structures on the DNA-112-Test and RNA-93-Test datasets. The results indicate that IKANbind outperforms other structure-based methods, indicating that IKANbind is also robust to proteins without available experimental structures.

The recently proposed AlphaFold3 (Abramson *et al.* 2024), which introduces a diffusion model and a unified molecular representation framework, achieves high-accuracy prediction of various protein-molecular complex structures, including protein–nucleic acid and small molecule complexes. To demonstrate the advantages of IKANbind over AlphaFolds on identifying NBRs, we conducted a performance comparison between IKANbind and AlphaFold3 on strictly independent test sets. Specifically, we selected protein entries released after the AlphaFold3 training date (30 September 2021) from the DNA-112-Test and RNA-93-Test datasets to avoid potential data leakage. AlphaFold3 takes single-chain protein sequences and nucleic acid sequences as input. We then annotated the binding residues in the proteins based on the protein–nucleic acid complex structures predicted by AlphaFold3, which adopts the same definition of binding residues as in the benchmark datasets. Table 4, available as supplementary data at *Bioinformatics* online presents the comparative results of AUC and AUPR. Compared to AlphaFold3, IKANbind achieved improvements of 0.269 in AUC and 0.131 in AUPR on the DNA-binding task, and improvements of 0.131 and 0.011 on the RNA-binding task, respectively. The results indicate that although AlphaFold3 has made significant progress in complex structure prediction, identifying binding residues solely based on predicted structures remains challenging. This may be related to the conformational flexibility observed in protein–nucleic acid interactions (Xu *et al.* 2025).

### 3.2 IKANbind learns to discriminate patterns of binding and non-binding residues

**Figure 2:**
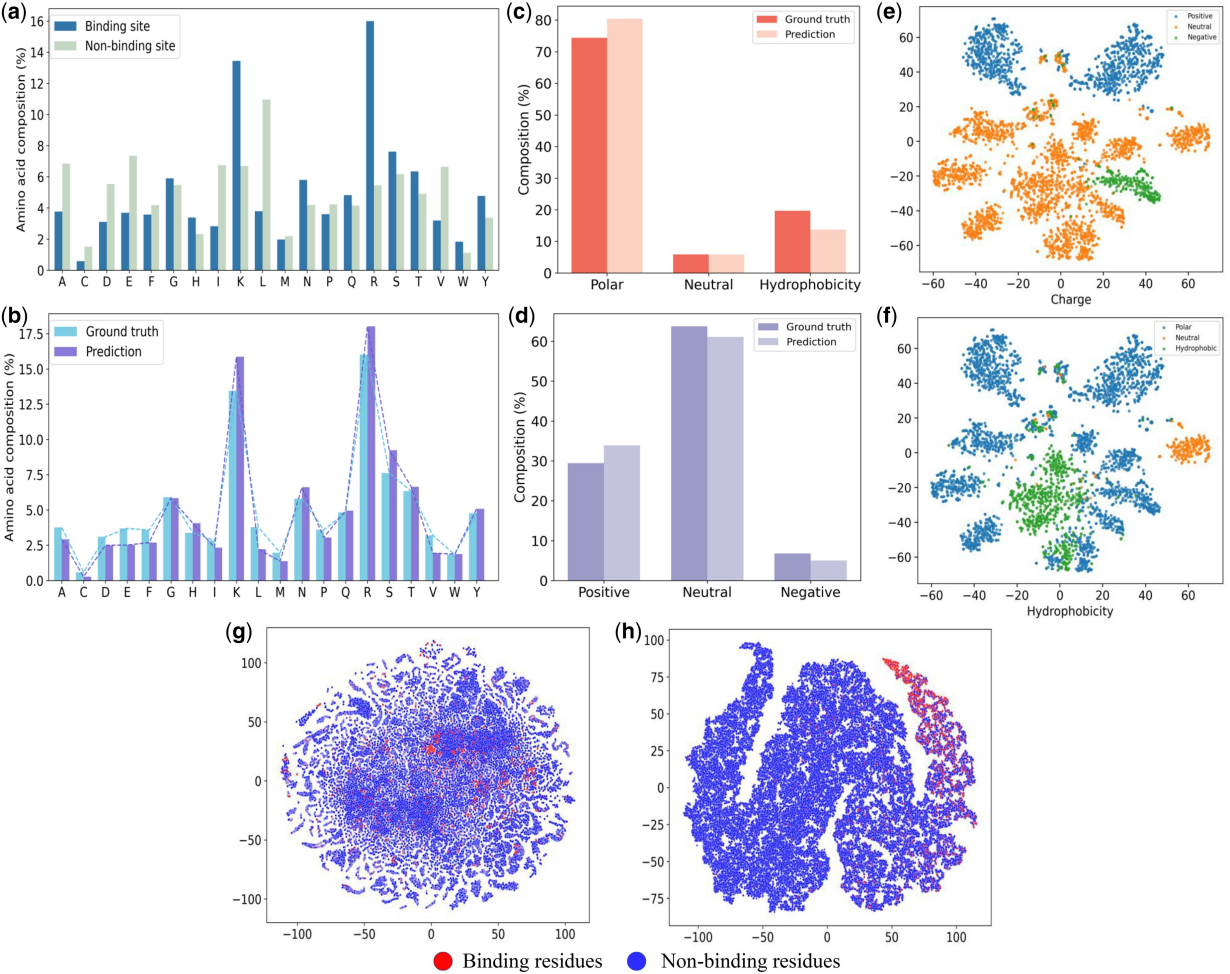
Analysis of amino acid composition and properties in DNA-180-Test. (a) Amino acid composition distribution of binding and non-binding sites. (b) Comparison of real and predicted binding site distributions. (c) and (d) Comparison of amino acid hydrophobicity and charge distributions between true and predicted binding sites. (e) and (f) Hydrophobicity and charge distributions of amino acids learned by the pLMs. (g) Sample distributions encoded by original embeddings. (h) Sample distributions encoded by IKANbind-learned feature vectors.

Proteins can specifically bind to nucleic acids through hydrogen bonds and hydrophobic interactions. This biological phenomenon is closely related to the amino acid composition and physicochemical properties of the protein. Therefore, we performed a statistical analysis of the amino acid composition and physicochemical properties at binding and non-binding sites on the DNA-180-Test dataset. We observed that lysine (K) and arginine (R) are highly enriched at the binding site in protein-DNA interactions (Fig. 2a). They are positively charged, which enables them to form stable bindings with the negatively charged nucleic acid backbone through electrostatic attraction (Jones *et al.* 1999). Additionally, we used the Kullback-Leibler (KL) divergence to measure the distribution distance between the predicted and true binding sites (Fig. 2b). The forward and reverse KL divergences were 0.021 and 0.023, respectively, approaching zero, indicating a high similarity between predicted and true distributions. This suggests that IKANbind can effectively capture the compositional features of amino acids.

In addition, we also analyzed the real and predicted hydrophobicity and charge distributions of amino acids in DNA-binding sites (Fig. 2c and d), which displays close alignment between predicted and true distributions. Regarding the contribution of pLM embeddings, we found that pLMs have learned substantial amino acid information, such as physicochemical properties. To validate it, we conducted a dimensionality reduction analysis of the pLM embeddings using t-SNE. We observed that different types of amino acid physicochemical properties form distinct clusters (Fig. 2e and f). Overall, the properties of the binding sites predicted by IKANbind are highly consistent with the true binding sites. The results on RNA-142-Test can be seen in the Fig. 2, available as supplementary data at *Bioinformatics* online.

**Figure 3:**
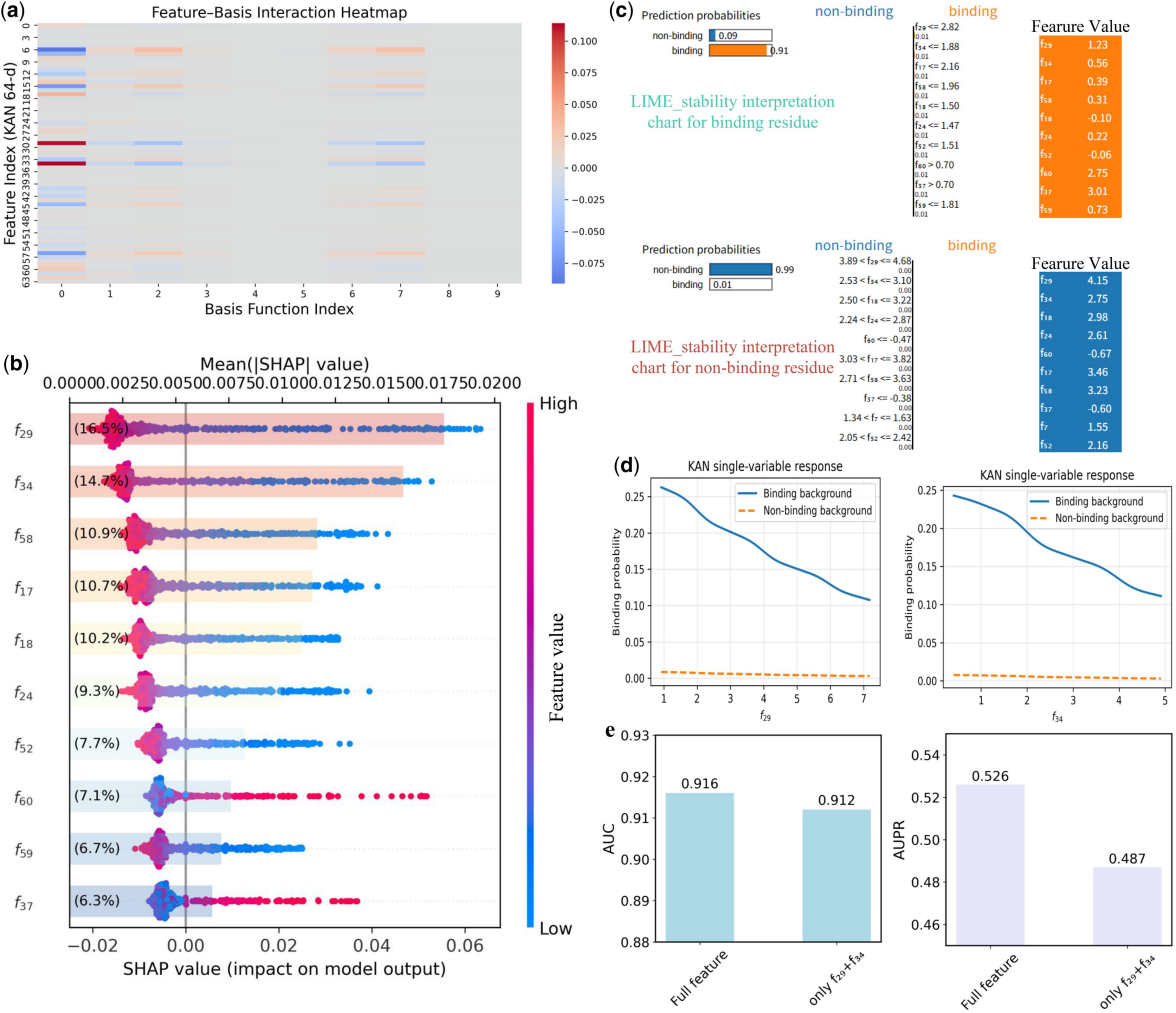
Analysis of nucleic acid binding sites based on discriminative features learned by IKANbind on the DNA-180-Test. (a) The degree of response between the latent representations learned by IKANbind and different basis functions, which indicates that darker colors correspond to greater contribution to the task. (b) SHAP summary plot. The bar chart shows the mean absolute SHAP value for each feature, reflecting its overall contribution to the task. (c) LIME_stability-based feature importance analysis. (d) Univariate perturbation analysis. Other features are fixed at their mean values, while only the target feature is continuously varied within its actual range. (e) Performance comparison between the key latent features and all latent features.

We further used t-SNE to evaluate the ability of IKANbind to learn discriminative representations. Figure 2g and h show the sample distributions encoded by the original embeddings (size 6229) and the learned latent feature vectors (size 64) on DNA-180-Test, respectively. In Fig. 2g, we can observe that binding and non-binding residues are randomly distributed and difficult to distinguish. In Fig. 2h, most binding residues tend to cluster together. The results indicate that the representations learned by IKANbind are more discriminative than the original features. The visualization results on RNA-142-Test, DNA-112-Test, and RNA-93-Test can be seen in Fig. 3, available as supplementary data at *Bioinformatics* online.

### 3.3 IKANbind captures the relevant features for nucleic acid-binding residues with KAN

As an alternative to MLP, which learn implicit neuron activations, KAN describes the nonlinear relationship between input and output through combinations of decomposable basis functions. Therefore, we designed two KAN schemes with different basis functions: B-spline (default) functions and a set of functions with explicit mathematical meaning. Partial mathematical expressions were then extracted from the last layer (shape: 64→1) for analysis (Table 3). Although the two schemes achieve comparable performance, the latter offers higher readability. Compared with the locally piecewise nature of B-splines, the symbolic basis functions possess clear mathematical meanings and global interpretability, which allows each parameter to correspond to an intuitive trend or pattern. In addition, we further extracted the weights of each basis function in the model and analyzed the degree of response between the features and different basis functions. In Fig. 3a, we found that these features exhibit the strongest response to tanh(*x*) (index 0), indicating that the model tends to use nonlinear mappings with saturation characteristics during NBR classification, thereby forming a threshold-like decision pattern. This phenomenon aligns with biological intuition in protein–nucleic acid interactions, where physicochemical properties such as amino acid charge often exert a decisive influence on binding behavior once they exceed a certain threshold.

**Table 3:** Comparison of KAN based on B-Spline function and symbolic function set.

Schemes	Dataset	AUC	AUPR	Expression
B-spline function	DNA-180-Test	0.913	0.518	Expression 1
	RNA-142-Test	0.837	0.336	Expression 2
Symbolic-function	DNA-180-Test	0.916	0.526	Expression 3
	RNA-142-Test	0.837	0.340	Expression 4
Expression 1	*y *= 0.003*B_1,1_(*f_1_*) + 0.002*B_1,2_(*f_1_*) − 0.002*B_1,7_(*f_1_*) + 0.002*B_2,1_(*f_2_*) + 0.003*B_2,2_(*f_2_*)			
Expression 2	*y *= 0.002*B_1,1_(*f_1_*) + 0.001*B_1,2_(*f_1_*) − 0.001*B_1,3_(*f_1_*) + 0.001*B_1,4_(*f_1_*) − 0.001*B_1,5_(*f_1_*)			
Expression 3	*y *= 0.026*tanh(π*f_0_) − 0.010*2*f_0_ + 0.012*tanh(π*f_3_) − 0.034*tanh(π*f_4_) + 0.011*sin(π*f_4_)			
Expression 4	*y *= 0.028*tanh(π*f_0_) − 0.010* sin(π*f_0_) − 0.011*2* *f_0 + _*0.014*tanh(π*f_3_) − 0.034*tanh(π*f_4_)			

KAN, due to its decomposable basis function weighting mechanism, can reveal the contribution of different features to the prediction task. As shown in Fig. 3a, features $f_{29}$ and $f_{34}$ exhibit more significant responses on the learned basis functions compared to other features, which indicates that they have a greater impact on the model’s prediction results. Considering that the feature-basis function responses are primarily based on KAN’s internal structure, there may be some randomness. Therefore, we further introduced two model-agnostic interpretability methods—Shapley Additive exPlanations (SHAP; Roth 1988) and Local Interpretable Model-Agnostic Explanations with stability (LIME_stability) (Visani *et al.* 2022)—to independently validate the feature importance and the stability of local decision-making from an external perspective. Details of SHAP and LIMI_stability can be seen in Section 2, available as supplementary data at *Bioinformatics* online. As illustrated in Fig. 3b, the mean SHAP values for features $f_{29}$ and $f_{34}$ are the highest, indicating that they contribute the most to the model’s predictions. Furthermore, we selected one binding residue and one non-binding residue from protein 6wc2_D and applied LIME_stability for local interpretability analysis. As visualized in Fig. 3c, features $f_{29}$ and $f_{34}$ exhibit a significant but opposing influence on these two classes of residues. We also conducted feature perturbation and ablation experiments. In the perturbation experiment, we fixed other features at their mean values and continuously varied the value of the target feature within its true range. We then plotted the response trend of the model output as the feature changed. Figure 3d displays the features that have the greatest impact on the prediction of binding residues. It is readily apparent that the values of features $f_{29}$ and $f_{34}$ are inversely proportional to the binding probability, which is validated by Fig. 3c. In the ablation experiments, we set other features to zero and retained only the key features to evaluate their impact on the model’s predictive performance. The results in Fig. 3e show that with only features $f_{29}$ and $f_{34}$, IKANbind still maintains promising performance. Based on the above analysis, we found that although deep learning has strong representation learning capabilities, key representations are still focused on a small number of features. Meanwhile, existing deep learning-based models struggle to accurately locate the specific representations containing critical information. IKANbind, based on the unique mechanism of KAN, can effectively identify the most crucial representations.

**Figure 4:**
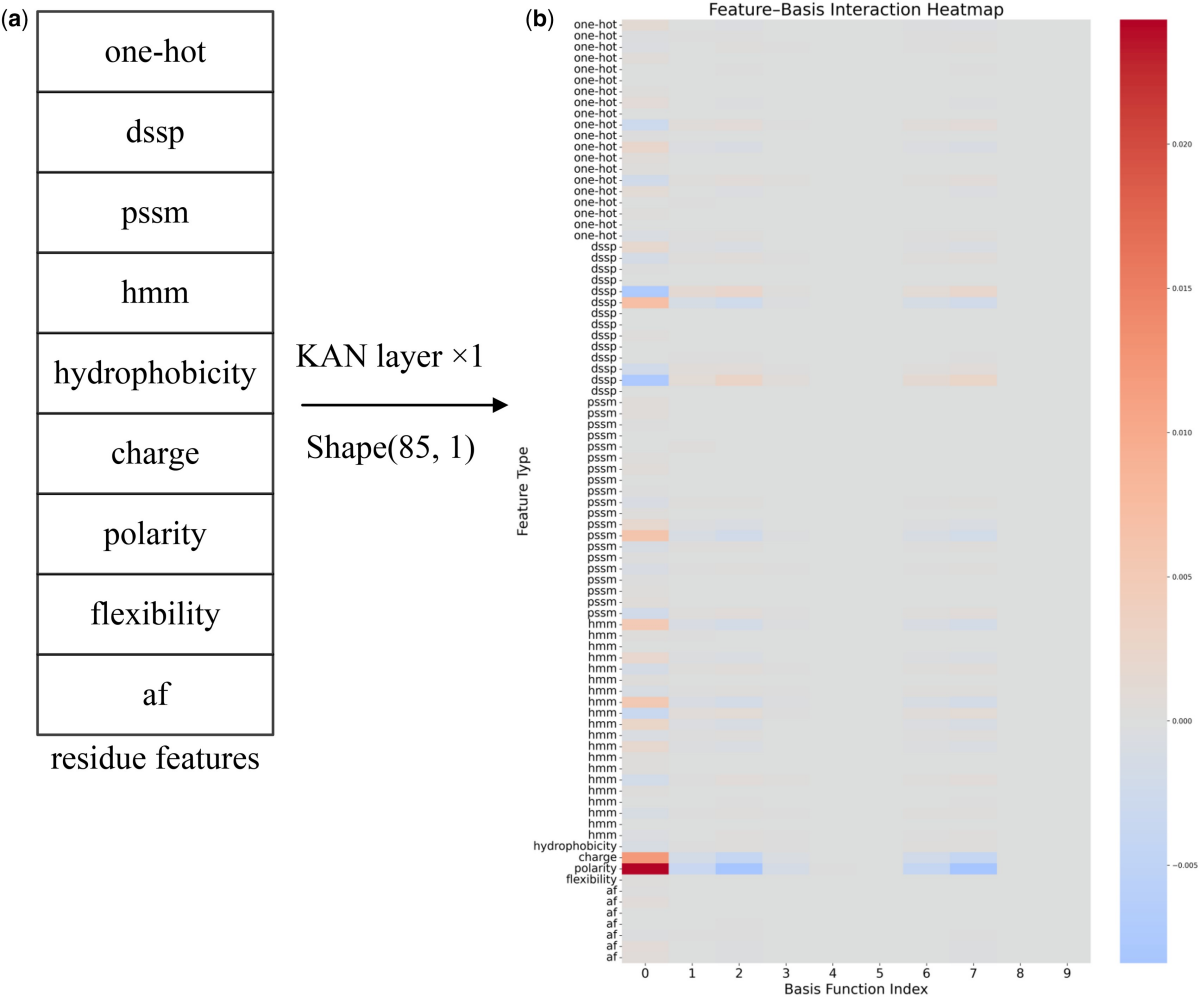
KAN was used to analyze the biological features associated with nucleic acid-binding residues. (a) Remove the protein language embeddings and input the remaining features into a single-layer KAN. (b) The response degree of different features and basis functions, which can reveal their importance to the prediction task.

However, the inherent “black-box” nature of deep neural networks means that the latent representations learned by the model are difficult to map back to the original features with clear biological meaning. To further validate KAN’s ability to identify key representations and evaluate the importance of biological features for NBR prediction, we directly input the protein sequences into a KAN network with only one layer for training and testing, with the other experimental settings consistent with IKANbind. Considering that pLMs can implicitly learn the physicochemical properties of amino acids (Fig. 2e and f), we removed the pLM embeddings from the residue feature space (Fig. 4a). Figure 4b shows that polarity and charge contribute more to the prediction of NBRs than other physicochemical properties and evolutionary information. The published study (Kulandaisamy *et al.* 2018) demonstrates that binding residues in protein-DNA complexes are mainly enriched with positively charged residues [such as lysine (K) and arginine (R), as observed in Fig. 2b]. At the same time, key residues that contribute to both binding and stability are composed of charged and polar amino acids. These results further prove the effectiveness of KAN in identifying key discriminative representations. However, it is important to note that this feature recognition mechanism is limited to the model level, rather than the biological level.

### 3.4 The importance of geometric information for NBRs prediction

To validate the importance of geometric information for NBRs prediction, we compared IKANbind with a geometry-agnostic baseline method, BiLSTM. To ensure a fair comparison, BiLSTM used the same residue features as IKANbind, with the MLP module replaced by KAN. As shown in Table 5, available as supplementary data at *Bioinformatics* online, IKANbind outperforms BiLSTM on all four independent test sets. Specifically, on the DNA-180-Test dataset, IKANbind improved AUC, AUPR, and MCC by approximately 2.0%, 14.3%, and 9.4%, respectively, compared to BiLSTM; On the RNA-142-Test dataset, the three metrics increased by about 1.7%, 16.0%, and 12.3%, respectively. In addition, IKANbind still outperformed BiLSTM on the DNA-112-Test and RNA-93-Test datasets, with improvements in AUC, AUPR, and MCC of 5.2%, 62.2%, 32.4% and 2.3%, 11.1%, 11.8%, respectively. The results indicate that geometric information is crucial for NBR prediction, and the hypergraph-based IKANbind excels at extracting spatial information on multi-residue interactions from predicted structures.

### 3.5 Case study

**Figure 5:**
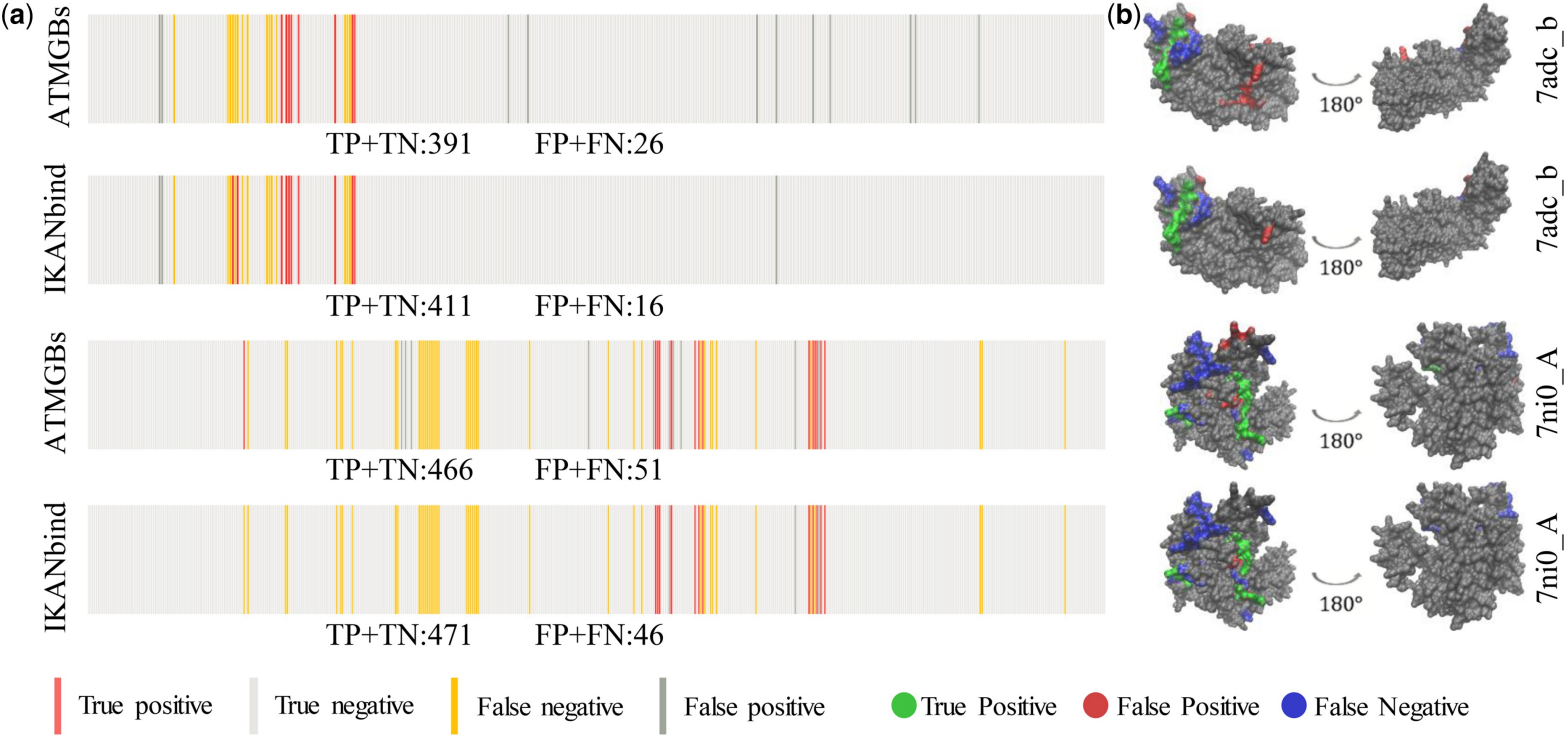
Case studies of IKANbind and the second best method ATMGBs for nucleic acid-binding residue prediction. (a) Residue position map with predicted and true binding residues for proteins 7adc_b and 7ni0_A. (b) 3D structure diagram of proteins 7adc_b and 7ni0_A.

In this section, we analyze two cases of IKANbind and the second-best method (ATMGBs) in the test set. Protein 7adc_b (417 residues) and 7ni0_A (517 residues) are selected in the DNA-180-Test and RNA-142-Test datasets. According to the residue position maps in Fig. 5a, IKANbind predicts 20 and 5 more residues on 7adc_b and 7ni0_A, respectively, compared to ATMGBs. Furthermore, the number of false positives predicted by IKANbind is significantly reduced. The illustrations of 7adc_b and 7ni0_A in Fig. 5b display the predicted residue distribution regions by ATMGBs and IKANbind. Specifically, the red and blue regions are fewer in the residue distribution predicted by IKANbind. The results suggest the effectiveness of IKANbind in predicting nucleic acid-binding residues.

### 3.6 Ablation studies on IKANbind

#### 3.6.1 Contribution of pLM embedding

**Figure 6:**
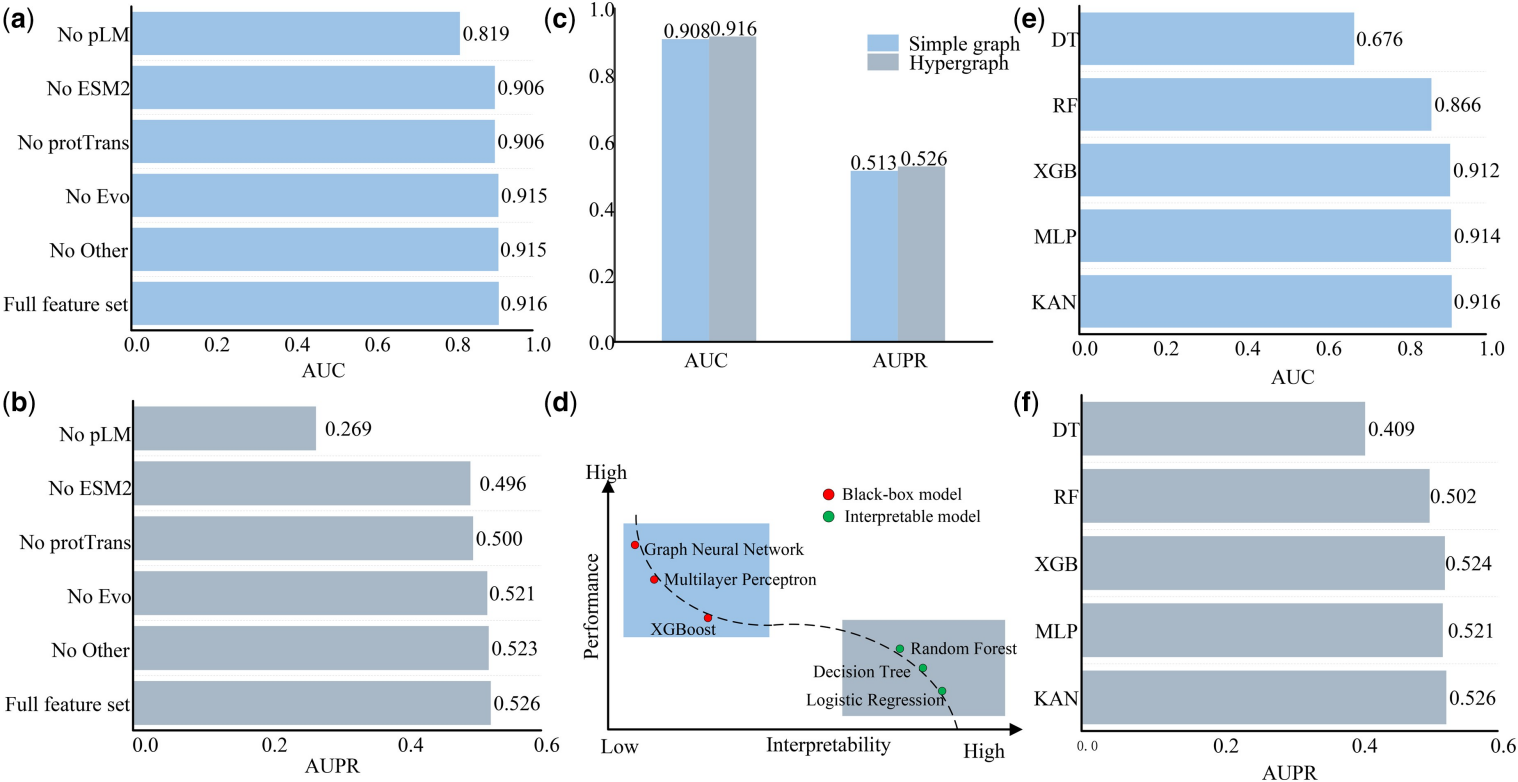
Ablation study of IKANbind on DNA-180-Test. (a) and (b) AUC and AUPR of IKANbind under different feature combinations. (c) The performance of hypergraph networks used in the full-fledged version of IKANbind is compared against the simple graph networks. (d) Trade-off between interpretability and performance. (e) and (f) AUC and AUPR of IKANbind under different classifiers.

IKANbind incorporates pretrained pLM (ESM2 and ProtTrans) embeddings as part of the node features. To evaluate the contribution of pLM embeddings, we conducted a feature ablation study, which sequentially removes pLM embeddings, evolutionary information, and other features from the complete IKANbind feature set. Figure 6a and b present the 5-CV performance of IKANbind with different feature combinations on DNA-180-Test. The results show that removing pLM embeddings (No pLM) causes the most significant performance drop, with AUC decreasing by 11.8%. In comparison, discarding evolutionary information or other features results in relatively minor performance decreases. For example, without evolutionary information (No Evo) or other features (No Other), the AUC decreased by only 0.1% and 0.1%, respectively. This significant performance gap highlights the importance of pLM embeddings in NBRs prediction. Furthermore, this also demonstrates that pLMs can effectively learn evolutionary and physicochemical information. The results for RNA-142-Test, DNA-112-Test, and RNA-93-Test can be seen in Table 6, available as supplementary data at *Bioinformatics* online.

#### 3.6.2 Contribution of hypergraph

To further investigate the effectiveness of the hypergraph structure in IKANbind, we conducted an ablation study to evaluate the impact of the hypergraph structure in IKANbind. Specifically, based onthe same threshold setting, we replaced the hyperedges in IKANbind with ordinary edges. Figure 6c compares the performance of the hypergraph (IKANbind full version) and the simple graph on DNA-180-Test. The results indicate that the hypergraph structure in the IKANbind consistently outperforms the simple graph structure. For example, the AUC (AUPR) for GCN was 0.908 (0.513), while IKANbind reached 0.916 (0.526). The results suggest that the hypergraph structure plays a positive role in NBRs prediction tasks. The results for RNA-142-Test, DNA-112-Test, and RNA-93-Test can be seen in Table 7, available as supplementary data at *Bioinformatics* online.

#### 3.6.3 Comparison of KAN with other classifiers

Figure 6d shows that there exists a trade-off between the model performance and interpretability (Karim *et al.* 2023), from logistic regression (traditional machine learning) to GNN (deep neural network). As an alternative to MLPs, KAN has attracted considerable attention due to its good performance and interpretability. To evaluate the performance of KAN, we compared it with Decision Tree, Random Forest, XGBoost (XGB), and MLP on DNA-180-Test. To ensure a fair comparison, we replaced only the classifier module of IKANbind. Given that machine learning does not have a backpropagation mechanism, we directly extract the corresponding features from the trained model as input for the machine learning classifier. Figure 6e and f present the comparison of KAN and other classifiers in AUC and AUPR. The results indicate that KAN outperforms other classifiers, achieving an AUC and AUPR of 0.916 and 0.526, respectively. The results on RNA-142-Test, DNA-112-Test, and RNA-93-Test can be seen in the Table 8, available as supplementary data at *Bioinformatics* online. We found that some machine learning classifiers, such as XGB, perform comparably to deep learning classifiers, which can be attributed to the powerful representation learning ability of the deep hypergraph neural network.

## 4 Discussion

This study presents IKANbind, a pLM-informed hypergraph neural network framework for accurate prediction of nucleic acid binding sites (NBRs). IKANbind utilizes hypergraphs to describe the interactions among multiple residues in the 3D structure of proteins. We demonstrate that IKANbind consistently outperforms state-of-the-art methods on the nucleic acid-binding site prediction tasks. Analysis of pLM reveals that it cannot only implicitly learn the physicochemical properties of amino acids (such as charge and hydrophobicity), but also reduce reliance on evolutionary information. In addition, the symbolic KAN, which utilizes a unique weighted mechanism of decomposable basis functions, can accurately identify the most critical features for the prediction task. We found that the charge and polarity of amino acids are more important for nucleic acid-binding residues than other physicochemical properties and evolutionary information.

To demonstrate the extendibility of IKANbind, we further investigated the performance of IKANbind in predicting other types of ligand-binding residues. We collected four benchmark ligand datasets from CLAPE and GraphBind, including antibody-antigen (AB), metal ions (${\text{Mn}}^{2+}$), and two biologically relevant molecules (ATP and HEME). Detailed information on ligand-binding residue datasets can be seen in the Table 9, available as supplementary data at *Bioinformatics* online. We followed the same training and evaluation strategy for the four ligands as for NBR. Figure 4, available as supplementary data at *Bioinformatics* online presents the comparison of IKANbind and the second-best method (ATMGBs) in AUC and MCC. The result shows that IKANbind consistently outperforms ATMGBs in four ligand prediction tasks. For example, in the AB task, IKANbind achieved AUC and MCC values of 0.912 and 0.494, which are higher than 0.908 and 0.491 of ATMGBs. These results suggest that IKANbind is equally applicable to other types of ligand-binding residue tasks.

Although IKANbind has the above advantages, several limitations remain. First, this work predicts nucleic acid-binding sites based solely on protein information, which lacks prior knowledge of the corresponding nucleic acid molecules. For example, adding additional information about the nucleic acid through DNA/RNA-related language models could be beneficial. Second, the dynamic conformations that arise during protein–nucleic acid complex formation may change the interactions between residues and affect the accessibility of binding sites. Considering the inclusion of conformational ensembles or binding-induced structural changes may further enhance the biological realism and prediction performance of the model. Finally, and most importantly, although the symbolic KAN can identify the most crucial features for NBRs, it cannot trace back whether these features originate from pLM embeddings or physicochemical properties, or others. This represents a pressing issue that needs to be addressed in current and future research: the “black-box” problem caused by the complex weights and activation functions in deep learning, which poses a significant challenge for interpretability at the biological level.

## Supplementary Material

btag433_Supplementary_Data

## Data Availability

IKANbind is freely available at https://github.com/yangfengzhuguet/IKANBind.
