# Supplementary material for: Protein–nucleic acid binding site prediction using interpretable Kolmogorov–Arnold networks with hypergraph representation learning
Source: Bioinformatics. 2026 Jun 20;42(7):btag433. doi: 10.1093/bioinformatics/btag433 (PMC13345923; doi:10.1093/bioinformatics/btag433)
Supplement: btag433_Supplementary_Data [file btag433_supplementary_data.docx]

**Supplementary Information for**

Protein–Nucleic Acid Binding Site Prediction Using Kolmogorov–Arnold Networks with Hypergraph Representation Learning

**AUTHORS**

Yangfeng Zhu^1+^, Guicong Sun^1+^, Weimin Zhu^2+^, Yongxian Fan^1^*，Zeheng Wu^1^, Xianchen Zheng^1^, and Xiaoyong Pan^2^*

^1^ School of Computer Science and InformationSecurity, Guilin University of ElectronicTechnology, Guilin 541004, China

^2^ Institute of Image Processing and Pattern Recognition, Shanghai Jiao Tong University, and Key Laboratory of System Control and Information Processing, Ministry of Education of China, Shanghai, 200240, China

^+^ These authors contributed equally to this work

^*^ Address correspondence to Y. Fan: yongxian.fan@gmail.com or X. Pan: [2008xypan@sjtu.edu.cn](mailto:2008xypan@sjtu.edu.cn)

**SUPPLEMENTARY DATA**

**Supplementary Sections**

- Section S1. Calculation formula for performance evaluation indicators.
- Section S2. SHAP and LIME_stability

**Supplementary Tables**

- Table S1: Overview of Node features
- Table S2: Summary of all the methods benchmarked in our work.
- Table S3: Comparison of IKANbind with structure-based methods on the DNA-112-Test and RNA-93-Test datasets (protein structures from experiments)
- Table S4: Comparison of IKANbind and Alphafold3 in AUC and AUPR
- Table S5: Comparison of IKANbind and BiLSTM
- Table S6: AUC and AUPR of IKANbind under different feature combinations on RNA-142-Test, DNA-112-Test, and RNA-93-Test.
- Table S7: The performance of hypergraph networks used in the full-fledged version of IKANbind is compared against the simple graph networks on RNA-142-Test, DNA-112-Test, and RNA-93-Test.
- Table S8: AUC and AUPR of different classifiers on RNA-142-Test, DNA-112-Test, and RNA-93-Test.
- Table S9: Summary of protein-ligand binding sites datasets

**Supplementary Figures**

- Figure S1. Per-protein GPU memory consumption comparison between IKANbind and EquiPNAs
- Figure S2. Analysis of amino acid composition and properties in RNA-142-Test.
- Figure S3. T-SNE visualization results on RNA-142-Test, DNA-112-Test, and RNA-93-Test.
- Figure S4. AUC and MCC of IKANbind and ATMGBs on four ligand test sets.

**Supplementary Sections**

- Section S1. Calculation formula for performance evaluation indicators.

$$\text{Accruary =}\frac{\text{TP+TN}}{\text{TP+TN+FP+FN}}$$

$$\text{Specificity =}\frac{\text{TN}}{\text{TN+FP}}$$

$$\text{Precision}=\frac{\text{TP}}{\text{TP}+\text{FP}}$$

$$\text{F1}=2\cdot\frac{\text{Precision}\times\text{Recall}}{\text{Precision+Recall}}$$

$$\text{MCC}=\frac{\text{TP}\times\text{TN}-\text{FN}\times\text{FP}}{\sqrt{\left(\text{TP}+\text{FP}\right)\times\left(\text{TP}+\text{FN}\right)\times\left(\text{TN}+\text{FP}\right)\times\left(\text{TN}+\text{FN}\right)}}$$

where true positives (TP) and true negatives (TN) denote the number of binding and non-binding sites identified correctly, and false positives (FP) and false negatives (FN) denote the number of incorrectly predicted binding and non-binding sites, respectively. In addition, the area under the receiver operating characteristic curve (AUC) and the area under the precision-recall curve (AUPR) are also utilized to evaluate model performance.

- Section S2. SHAP and LIME_stability

SHAP uses the SHAP value to measure the impact of the characteristics of a complex model. The SHAP value is defined as the weighted average of the marginal contributions [[1](https://bmcbioinformatics.biomedcentral.com/articles/10.1186/s12859-023-05456-0#ref-CR21)]. It can be used to explain any type of predictive model for classification or regression.

LIME, a black-box model interpretation method, interprets the model by providing a model that behaves very similarly to the original model [2]. It approximates the black box model f by using a simple function g around a point x, where g must belong to the class of interpretable models G. Each model corresponds to a specific input point x, only around x are the predictions of the interpretable model guaranteed to be very close to the black box model. This property determines the ability of LIME to act as a local interpretable tool.

Each time LIME is used, it generates new data points that follow the same distribution but differ in different applications. Due to the random nature of sampling, using different issues, different interpretable models may be obtained, thus obtaining different interpretations for the selected individuals [3]. To avoid uncertainty in model interpretation, we use an enhanced LIME model with a statistical stability index in this study [3] (<https://github.com/giorgiovisani/LIME_stabilitly>), which assesses the LIME by developing a complementary pair of indices for stability: the Variable Stability Index (VSI) and the System Stability Index (CSI). The VSI index is used to check whether different LIMEs return the same variables as explanations, and the CSI index controls whether the coefficients of each variable can be considered equal under repeated LIME calls.

**Supplementary Tables**

- Table S1: Overview of Node features

| Feature[shape] | Description |
| --- | --- |
| One-hot encoding[L,20] | One-hot encodings of 20 amino acid residue types |
| HMM[L,20] | Normalized Hidden Markov Models Matrix |
| PSSM[L,20] | Normalized position-specific scoring matrix |
| AF[L,7] | Atomic features of residues |
| ESM-2[L,5120] | PLM embeddings from ESM-2 with 15B |
| ProtTrans[L,1024] | PLM embeddings from ProtTrans |
| DSSP[L,14] | Secondary structure profiles of proteins |
| PP[L,4] | Physicochemical properties of residue |

- Table S2：Summary of all the methods studied in our work.

| Approachs | Techniques | Features | Ligands | Year |
| --- | --- | --- | --- | --- |
| COACH-D | COFACTOR[4], FINDSITE[5], TM-SITE[6], S-SITE, ConCavity[7] | Predicted protein structures, Evolutionary Sequence Conservation | DNA/RNA | 2018 |
| NucBind | SVMnuc, COACH-D | Features used in COACH-D and SVMnuc | DNA/RNA | 2019 |
| SVMnuc | SVM | PSI-BLAST profile, PSIPRED profile, HHblits profile | DNA/RNA | 2019 |
| DNAPred | SVM | PSSM^a^, PSS^b^,  PRSA^c^, AAFD-BN^d^ | DNA | 2019 |
| GraphBind | GNN | pseudo-positions^e^, atomic features of residues, secondary structure profiles, evolutionary conversation profiles | DNA/RNA | 2021 |
| GLMSite | PLM, GVP-GNN | Residue orientation^i^, DSSP, ProtTrans, Positional embedding, Distance encoding, Edge vector features | DNA/RNA | 2023 |
| CLAPE | PLM, CNN | ProtBERT | DNA | 2024 |
| ATMGBs | Attention Map, GCN | ProtT5, ESM1b, physicochemical properties | DNA/RNA | 2025 |
| EquiPNAs | EGNN | PSSM, MSA, ESM-2, one-hot, SS^f^, RSA^g^, Local geometry^h^, Residue orientation^i^, Relative residue positioning^j^, Residue virtual surface area^k^, Contact count^l^ | DNA/RNA | 2024 |

^a^position-specific scoring matrix, ^b^predicted secondary structure, ^c^predicted relative solvent accessibility, ^d^amino acid frequency difference between binding and nonbinding, ^e^the centroid of a residue including both backbone and side-chain atoms of the residue, ^f^One-hot encodings of 3- and 8-state secondary structure, ^g^One-hot encodings of 2- and 8-state relevant solvent accessibility, ^h^Cosine angle between the C=O of consecutive residues, normalized values of virtual bond and torsion angles, and normalized peptide backbone torsion angles, ^i^Unit vectors pointing towards the directions of $C_{\alpha}^{(i+1)}-C_{\alpha}^{i}$, $C_{\alpha}^{(i-1)}-C_{\alpha}^{i}$ and $C_{\beta}^{(i)}-C_{\alpha}^{i}$, ^j^Two types of relative positional features for the ith residue: (i) inverse of i representing the relative sequence position, and (ii) inverse of the Euclidean distance of $C_{\alpha}$ atom from the centroid representing the relative spatial positioning, ^k^Virtual surface area of the conceptual convex hull constructed by the atoms in a residue, ^l^The number of spatial neighbors of each residue.

- Table S3: Comparison of IKANbind with structure-based methods on the DNA-112-Test and RNA-93-Test datasets (protein structures from experiments).

| Dataset | Method | ACC | PRE | RECALL | F1 | MCC | AUC | AUPR |
| --- | --- | --- | --- | --- | --- | --- | --- | --- |
| DNA-112-Test | GLMSite | 0.948 | 0.421 | 0.546 | 0.475 | 0.453 | 0.917 | 0.453 |
|  | EquiPNAs | 0.943 | 0.393 | **0.610** | 0.478 | 0.461 | 0.932 | 0.461 |
|  | IKANbind | **0.955** | **0.472** | 0.515 | **0.493** | **0.469** | **0.933** | 0.**473** |
| RNA-93-Test | GLMSite | **0.899** | 0.323 | 0.412 | 0.362 | 0.310 | 0.822 | 0.298 |
|  | EquiPNAs | 0.878 | 0.304 | **0.569** | 0.396 | 0.356 | 0.850 | 0.357 |
|  | IKANbind | 0.891 | **0.327** | 0.529 | **0.404** | **0.360** | **0.862** | **0.361** |

- Table S4：Comparison of IKANbind and Alphafold3 in AUC and AUPR.

|  | DNA | | RNA | |
| --- | --- | --- | --- | --- |
|  | AUC | AUPR | AUC | AUPR |
| Alphafold3 | 0.662 | 0.308 | 0.729 | 0.348 |
| IKANbind | 0.931 | 0.439 | 0.860 | 0.359 |

- Table S5: Comparison of IKANbind and BiLSTM.

| **Dataset** | **Method** | **ACC** | **PRE** | **REC** | **F1** | **MCC** | **AUC** | **AUPR** |
| --- | --- | --- | --- | --- | --- | --- | --- | --- |
| DNA-180-Test | BiLSTM | 0.918 | 0.406 | 0.561 | 0.471 | 0.434 | 0.898 | 0.460 |
|  | IKANbind | 0.923 | 0.434 | 0.611 | 0.508 | 0.475 | 0.916 | 0.526 |
| RNA-142-Test | BiLSTM | 0.863 | 0.293 | 0.461 | 0.356 | 0.300 | 0.823 | 0.293 |
|  | IKANbind | 0.893 | 0.350 | 0.445 | 0.392 | 0.337 | 0.837 | 0.340 |
| DNA-112-Test | BiLSTM | 0.930 | 0.304 | 0.500 | 0.378 | 0.355 | 0.887 | 0.291 |
|  | IKANbind | 0.953 | 0.457 | 0.535 | 0.493 | 0.470 | 0.933 | 0.472 |
| RNA-93-Test | BiLSTM | 0.868 | 0.275 | 0.542 | 0.365 | 0.321 | 0.842 | 0.324 |
|  | IKANbind | 0.889 | 0.323 | 0.534 | 0.403 | 0.359 | 0.861 | 0.360 |

- Table S6: AUC and AUPR of IKANbind under different feature combinations on RNA-142-Test, DNA-112-Test, and RNA-93-Test.

| Feature | RNA-142-Test | | DNA-112-Test | | RNA-93-Test | |
| --- | --- | --- | --- | --- | --- | --- |
|  | AUC | AUPR | AUC | AUPR | AUC | AUPR |
| No pLM | 0.738 | 0.226 | 0.856 | 0.265 | 0.756 | 0.231 |
| No ESM2 | 0.825 | 0.318 | 0.924 | 0.441 | 0.831 | 0.319 |
| No protTrans | 0.826 | 0.321 | 0.926 | 0.449 | 0.853 | 0.344 |
| No evo | 0.837 | 0.341 | 0.933 | 0.471 | 0.859 | 0.359 |
| No other | 0.836 | 0.340 | 0.932 | 0.473 | 0.857 | 0.355 |
| Full feature set | 0.837 | 0.340 | 0.933 | 0.472 | 0.861 | 0.360 |

- Table S7: The performance of hypergraph networks used in the full-fledged version of IKANbind is compared against the simple graph networks on RNA-142-Test, DNA-112-Test, and RNA-93-Test.

| Feature | RNA-142-Test | | DNA-112-Test | | RNA-93-Test | |
| --- | --- | --- | --- | --- | --- | --- |
|  | AUC | AUPR | AUC | AUPR | AUC | AUPR |
| Simple graph | 0.836 | 0.336 | 0.929 | 0.456 | 0.848 | 0.347 |
| Hypergraph | 0.837 | 0.340 | 0.933 | 0.472 | 0.861 | 0.360 |

- Table S8: AUC and AUPR of different classifiers on RNA-142-Test, DNA-112-Test, and RNA-93-Test.

| classifier | RNA-142-Test | | DNA-112-Test | | RNA-93-Test | |
| --- | --- | --- | --- | --- | --- | --- |
|  | AUC | AUPR | AUC | AUPR | AUC | AUPR |
| DT | 0.607 | 0.295 | 0.696 | 0.384 | 0.602 | 0.276 |
| RF | 0.787 | 0.309 | 0.889 | 0.446 | 0.798 | 0.299 |
| XGB | 0.833 | 0.348 | 0.929 | 0.459 | 0.855 | 0.340 |
| MLP | 0.834 | 0.339 | 0.934 | 0.470 | 0.857 | 0.357 |
| KAN | 0.837 | 0.340 | 0.933 | 0.472 | 0.861 | 0.360 |

- Table S9: Summary of protein-ligand binding sites datasets

| Type | Dataset | Nproteina | Nposb | Nnegc |
| --- | --- | --- | --- | --- |
| AB | AB_Train_1011 | 1011 | 15749 | 189519 |
|  | AB_Test_259 | 259 | 3755 | 50890 |
| ATP | ATP_Train_388 | 388 | 5657 | 142086 |
|  | ATP_Test_41 | 41 | 674 | 14159 |
| Mn2+ | MN_Train_440 | 440 | 1931 | 150229 |
|  | MN_Test_144 | 144 | 612 | 50838 |
| HEME | HEM_Train_175 | 175 | 3851 | 44477 |
|  | HEM_Test_96 | 96 | 2012 | 26341 |

**Supplementary Figures**


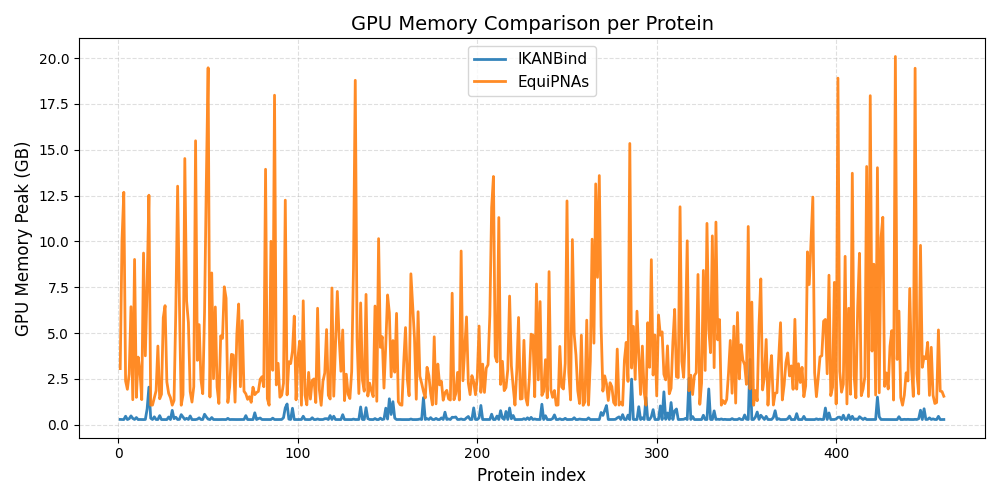


- Figure S1. Per-protein GPU memory consumption comparison between IKANBind and EquiPNAs.


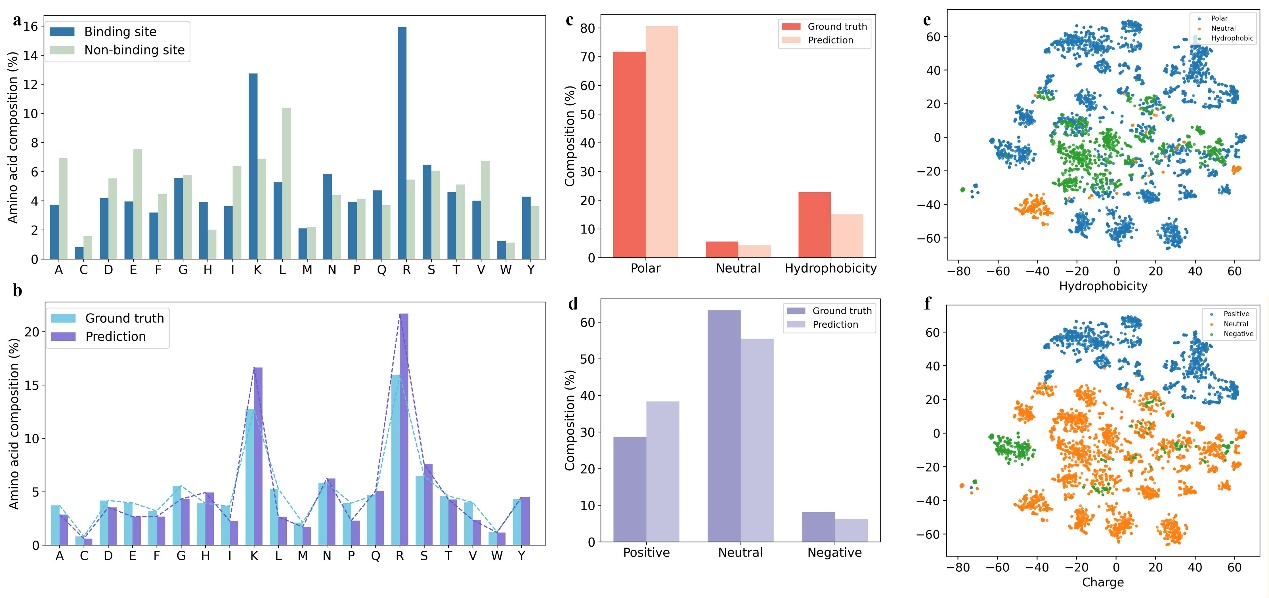


- Figure S2. Analysis of amino acid composition and properties in RNA-142-Test. **a** Amino acid composition distribution of binding and non-binding sites. **b** Comparison of real and predicted binding site distributions. **c**, **d** Comparison of amino acid hydrophobicity and charge distributions between true and predicted binding sites. **e**, **f** Hydrophobicity and charge distributions of amino acids learned by the PLMs.


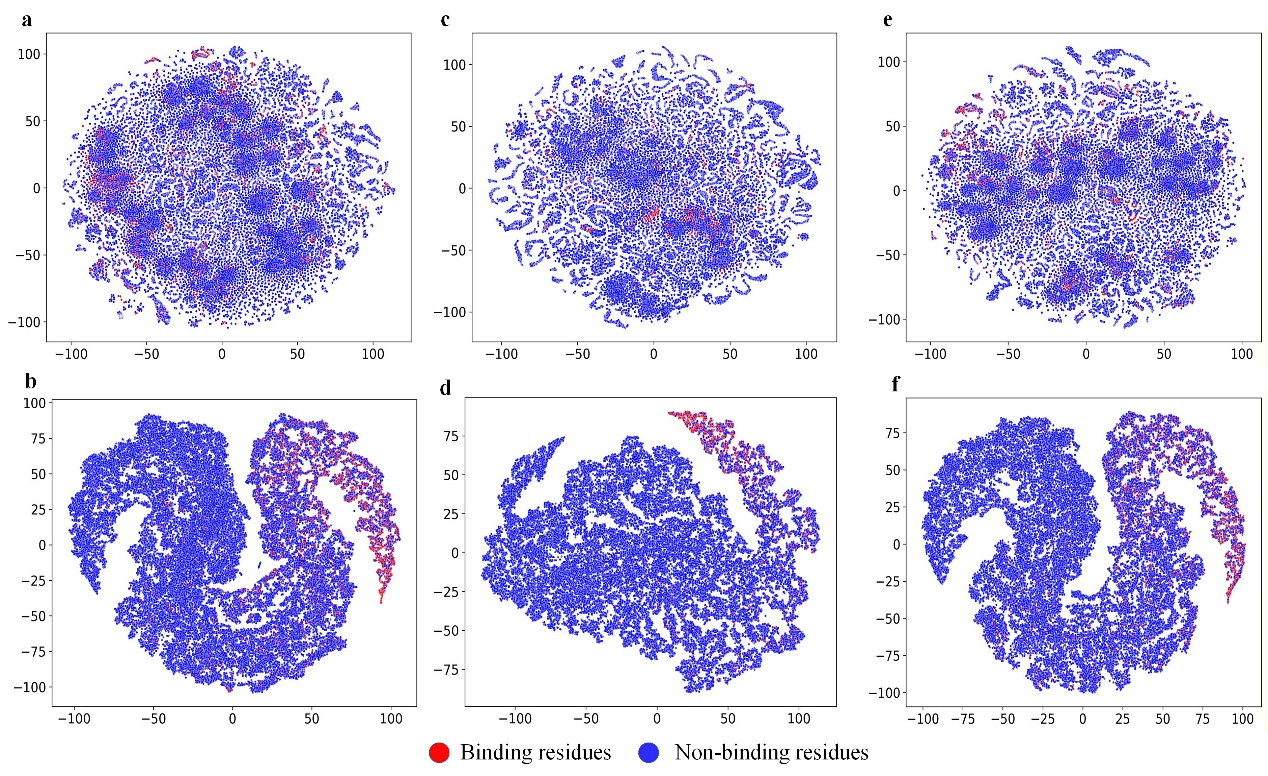


- Figure S3. T-SNE visualization results on RNA-142-Test, DNA-112-Test, and RNA-93-Test. Original embeddings (**a**) and IKANbind -learned feature vectors (**b**) on RNA-142-Test. Original embeddings (**c**) and IKANbind -learned feature vectors (**d**) on DNA-112-Test. Original embeddings (**e**) and IKANbind -learned feature vectors (**f**) on RNA-93-Test.


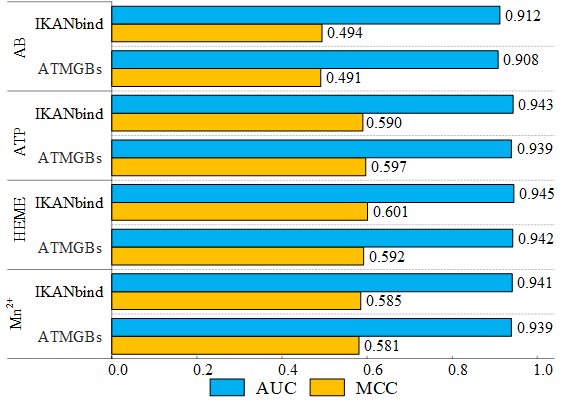


- Figure S4. AUC and MCC of IKANbind and ATMGBs on four ligand test sets.

**Reference**

[1] Štrumbelj E, Kononenko I. Explaining prediction models and individual predictions with feature contributions. Knowl Inf Syst. 2014;41:647–65.

[2] Ribeiro MT, Singh S, Guestrin C. “Why Should I Trust You?”: Explaining the predictions of any classifier. In: Proceedings of the 22nd ACM SIGKDD international conference on knowledge discovery and data mining. San Francisco: ACM; 2016. p. 1135–44.

[3] Visani G, Bagli E, Chesani F, Poluzzi A, Capuzzo D. Statistical stability indices for LIME: obtaining reliable explanations for machine learning models. J Oper Res Soc. 2022;73:91–101.

[4] Roy A, Yang J, Zhang Y. COFACTOR: an accurate comparative algorithm for structure-based protein function annotation, Nucleic acids research 2012;40:W471-W477.

[5] Brylinski M, Skolnick J. A threading-based method (FINDSITE) for ligand-binding site prediction and functional annotation, Proceedings of the National Academy of Sciences 2008;105:129-134.

[6] Yang J, Roy A, Zhang Y. Protein–ligand binding site recognition using complementary binding-specific substructure comparison and sequence profile alignment, Bioinformatics 2013;29:2588-2595.

[7] Capra JA, Laskowski RA, Thornton JM et al. Predicting protein ligand binding sites by combining evolutionary sequence conservation and 3D structure, PLoS computational biology 2009;5:e1000585.
